# Publisher Correction: Controllability governs the balance between Pavlovian and instrumental action selection

**DOI:** 10.1038/s41467-020-17420-0

**Published:** 2020-07-08

**Authors:** Hayley M. Dorfman, Samuel J. Gershman

**Affiliations:** Department of Psychology and Center for Brain Science, Harvard University, Northwest Lab Building, 52 Oxford Street, Cambridge, MA 02138 USA

**Keywords:** Cognitive neuroscience, Computational neuroscience, Learning algorithms, Learning and memory, Reward

Correction to: *Nature Communications* 10.1038/s41467-019-13737-7, published online 20 December 2019.

The original version of this Article contained an error in the table in Figure 2b that was inadvertently introduced during production. The last row in the first column of the table should read “High Control Decoy” as opposed to “Low Control Decoy.”

This error has been corrected in both the PDF and HTML versions of the Article.

