# Supplementary material for: Controllability governs the balance between Pavlovian and instrumental action selection
Source: Nat Commun. 2019 Dec 20;10:5826. doi: 10.1038/s41467-019-13737-7 (PMC6925275; doi:10.1038/s41467-019-13737-7)
Supplement: Supplementary file 1 — Supplementary Information [file 41467_2019_13737_MOESM1_ESM.pdf]

SUPPLEMENTARY INFORMATION

Controllability Governs the Balance Between Pavlovian and Instrumental Action Selection

Dorfman, et al.

Supplementary Methods

The Bayesian model used in this study was derived as follows. The generative model of an uncontrollable environment assumed by the Pavlovian system is given by:

$$\theta_s \sim Beta\left(\theta_0 \frac{\eta_0}{2}, (1-\theta_0)\frac{\eta_0}{2}\right) \qquad (1)$$
$$r|s \sim Bernouli(\theta_s)$$

where θ_s is the reward probability for stimulus *s*, θ_0 is the mean of the prior, η_0 is a parameter controlling the dispersion of the prior, and *r* is the reward outcome on a particular trial (all reward outcomes are assumed to be independently and identically distributed). The generative model of a controllable environment assumed by the instrumental system is essentially the same, except conditioned on action, *a*:

$$\theta_{sa} \sim Beta\left(\theta_0 \frac{\eta_0}{2}, (1-\theta_0)\frac{\eta_0}{2}\right) \qquad (2)$$
$$r|s,a \sim Bernouli(\theta_{sa})$$

For both systems, the posterior conditional on the stimulus-action-reward history $\mathcal{D}$ is a Beta distribution with the same functional form as the prior, shown here for the Pavlovian system:

$$P(\theta_s|\mathcal{D}, m = uncontrollable) = Beta(\hat{\theta}_s \frac{\eta_s}{2}, (1-\hat{\theta}_s)\frac{\eta_s}{2}) \qquad (3)$$
$$\hat{\theta}_s = \frac{\eta_0 + N_s}{\eta_s}$$
$$\eta_s = \eta_0 + T_s$$

where *m* indexes the assumed environment (uncontrollable for the Pavlovian system, controllable for the instrumental system), N_s is the number of times stimulus *s* was paired with reward, and T_s is the number of times stimulus *s* was presented. The equations for the instrumental system are identical except for conditioning on both stimuli and actions. Through simple algebraic manipulation, the update for the posterior mean $\hat{\theta}_s$ can be expressed as a recursive learning rule (Eq. 2 in the main text).

The posterior over generative models is given by:

$$P(m = uncontrollable|\mathcal{D}) \propto P(\mathcal{D}|m = uncontrollable)P(m = uncontrollable). \qquad (4)$$

The marginal likelihood is an integral over the latent parameters:

$$P(\mathcal{D}|m = uncontrollable) = \int P(\mathcal{D}|m = uncontrollable, \theta_s)P(\theta_s)d\theta_s \qquad (5)$$
$$= \frac{B(\hat{\theta}_s \frac{\eta_s}{2}, (1-\hat{\theta}_s)\frac{\eta_s}{2})}{B(\theta_0 \frac{\eta_0}{2}, (1-\theta_0)\frac{\eta_0}{2})}$$

where B denotes the beta function. The beta function can be expressed recursively:

$$B(x+1,y) = B(x,y)\frac{x}{x+y} \quad (6)$$

$$B(x,y+1) = B(x,y)\frac{y}{x+y}$$

Applying these recursions to the log posterior odds over m, one obtains the update rule for L in the main text (Eq. 6).

Supplementary Figures

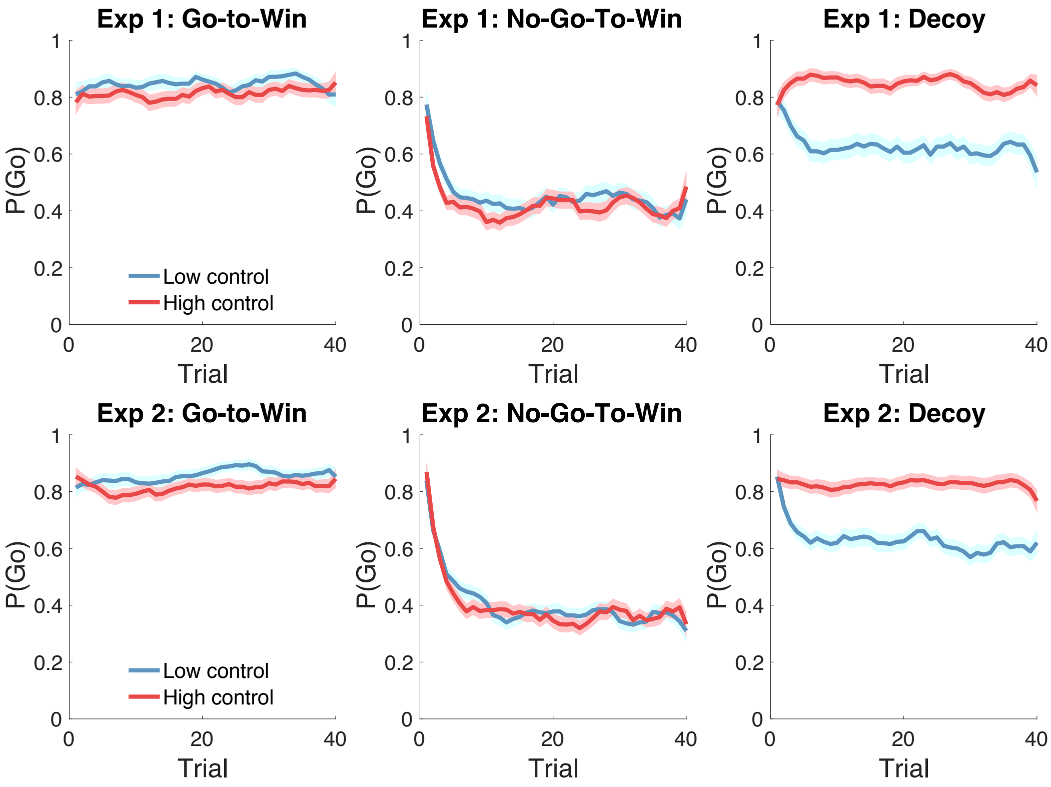

Supplementary Figure 1. Probability of Go response across trials for each experimental condition and stimulus, smoothed with a 5-trial moving average. Error bars show standard error of the mean.

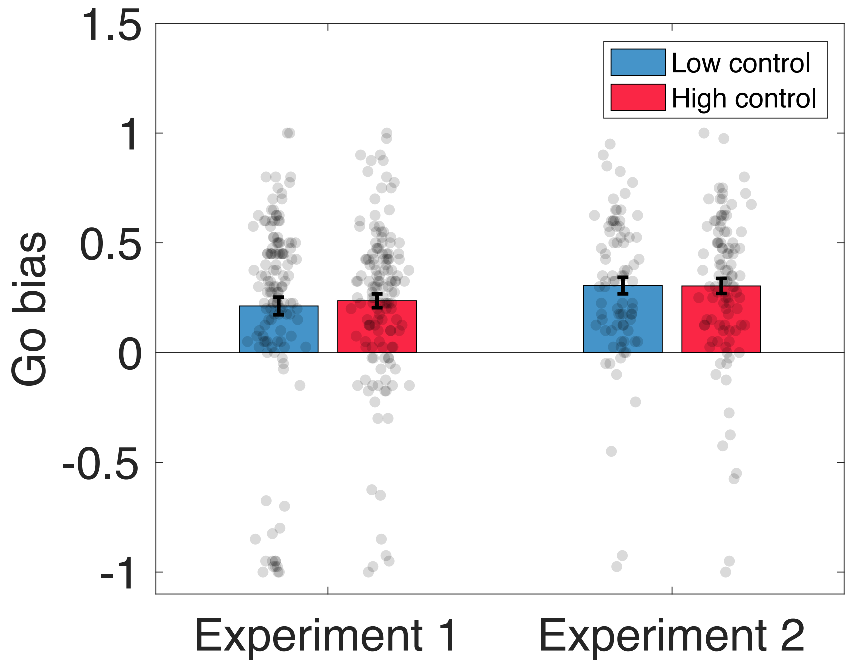

Supplementary Figure 2. Go bias for Experiment 1 (N = 271) and Experiment 2 (N = 183) when no subjects are excluded. For both experiments, there is a strong Go bias but no effect of controllability. Error bars show standard error of the mean.

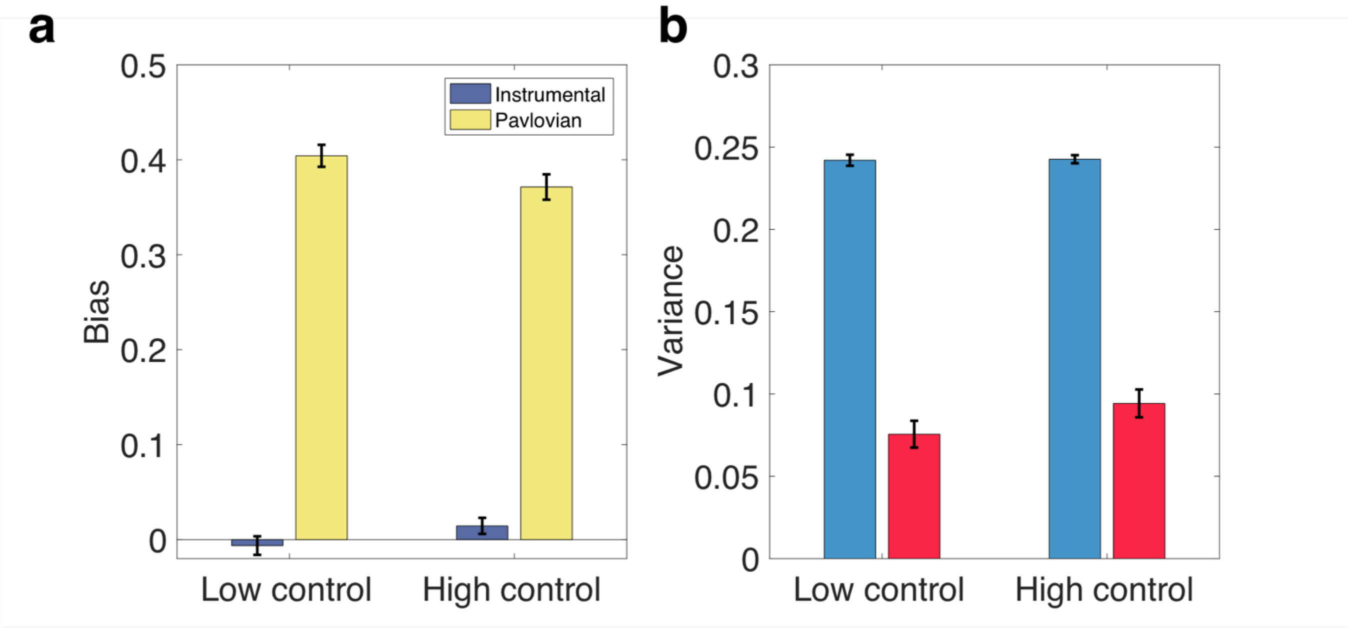

Supplementary Figure 3. Bias-variance analysis for purely Pavlovian ($w = 1$) and purely instrumental ($w = 0$) agents. Error bars show standard error of the mean.

Supplementary Table

	Inverse temperature	Prior mean (instrumental)	Prior confidence (instrumental)	Prior mean (Pavlovian)	Prior confidence (Pavlovian)
Experiment 1	11.3949	0.3774	33.3939	0.4848	34.4666
Experiment 2	10.4635	0.2189	31.5312	0.4628	29.4448

Supplementary Table 1. Average parameter estimates for the adaptive model.
