# Supplementary material for: Controllability governs the balance between Pavlovian and instrumental action selection
Source: Nat Commun. 2019 Dec 20;10:5826. doi: 10.1038/s41467-019-13737-7 (PMC6925275; doi:10.1038/s41467-019-13737-7)
Supplement: Supplementary file 3 — Reporting Summary [file 41467_2019_13737_MOESM3_ESM.pdf]

nature research

Corresponding author(s): Hayley Dorfman, Samuel Gershman

Last updated by author(s): November 11, 2019

Reporting Summary

Nature Research wishes to improve the reproducibility of the work that we publish. This form provides structure for consistency and transparency in reporting. For further information on Nature Research policies, see Authors & Referees and the Editorial Policy Checklist .

Statistics

For all statistical analyses, confirm that the following items are present in the figure legend, table legend, main text, or Methods section.

n/a	Confirmed	
[ ]	[x]	The exact sample size (*n*) for each experimental group/condition, given as a discrete number and unit of measurement
[ ]	[x]	A statement on whether measurements were taken from distinct samples or whether the same sample was measured repeatedly
[ ]	[x]	The statistical test(s) used AND whether they are one- or two-sided<br>*Only common tests should be described solely by name; describe more complex techniques in the Methods section.*
[ ]	[x]	A description of all covariates tested
[x]	[ ]	A description of any assumptions or corrections, such as tests of normality and adjustment for multiple comparisons
[ ]	[x]	A full description of the statistical parameters including central tendency (e.g. means) or other basic estimates (e.g. regression coefficient) AND variation (e.g. standard deviation) or associated estimates of uncertainty (e.g. confidence intervals)
[ ]	[x]	For null hypothesis testing, the test statistic (e.g. *F*, *t*, *r*) with confidence intervals, effect sizes, degrees of freedom and *P* value noted<br>*Give P values as exact values whenever suitable.*
[x]	[ ]	For Bayesian analysis, information on the choice of priors and Markov chain Monte Carlo settings
[x]	[ ]	For hierarchical and complex designs, identification of the appropriate level for tests and full reporting of outcomes
[ ]	[x]	Estimates of effect sizes (e.g. Cohen's *d*, Pearson's *r*), indicating how they were calculated

Our web collection on statistics for biologists contains articles on many of the points above.

Software and code

Policy information about availability of computer code

Data collection	The experiment was coded in jsPsych, version 6.0.5.
Data analysis	Data analysis was conducted in Matlab and R.

For manuscripts utilizing custom algorithms or software that are central to the research but not yet described in published literature, software must be made available to editors/reviewers. We strongly encourage code deposition in a community repository (e.g. GitHub). See the Nature Research guidelines for submitting code & software for further information.

Data

Policy information about availability of data

All manuscripts must include a data availability statement. This statement should provide the following information, where applicable:

- Accession codes, unique identifiers, or web links for publicly available datasets
- A list of figures that have associated raw data
- A description of any restrictions on data availability

The experiment code is posted at: https://github.com/hayleydorfman/pavlovian-instrumental-arbitration. Data analysis (computational modeling and statistics) and data are available at: https://github.com/sjgershm/GoNogo-control.

Field-specific reporting

Please select the one below that is the best fit for your research. If you are not sure, read the appropriate sections before making your selection.

[ ] Life sciences [x] Behavioural & social sciences [ ] Ecological, evolutionary & environmental sciences

Behavioural & social sciences study design

All studies must disclose on these points even when the disclosure is negative.

Study description	This is a quantitative experimental study assessing behavior in a Go/NoGo paradigm with a control manipulation. The study also includes a computational model that presents a possible mechanism for Pavlovian-instrumental arbitration.
Research sample	This is a behavioral study of human adults collected online via Amazon Mechanical Turk. Sample sizes were chosen to exceed similar, previous work (see manuscript for citations). Experiment 1: N = 271, Experiment 2: N = 183.
Sampling strategy	Participants from Experiment 1 were recruited randomly, and randomly assigned to an experimental condition. Participants for Experiment 2 were recruited from an existing pool of Amazon Mechanical Turk workers. These workers have completed previous experiments for our lab and expressed interest in being re-contacted for additional study opportunities and completed both experimental conditions (randomized order).
Data collection	Data was collected online via Amazon Mechanical Turk (computer). Researchers were not blinded to the hypotheses.
Timing	Experiment 1: 6/18/19-6/21/19 (this sample was recollected due to initial error - see previous revision letter). Experiment 2: 1/14/19-1/17/19
Data exclusions	Participants were excluded for inaccuracy. Specifically, if participants made the incorrect action (either a button press for a No-Go trial, or the absence of a button press for a Go trial) for > /= 50% of all trials, they were excluded from analyses (predetermined criteria). We also excluded any subjects that performed less than 30% on any one condition (criteria determined post-hoc when researchers noticed this was a problem in the data). This left a total of 185 accurate participants for Experiment 1 and 129 accurate participants for Experiment 2.
Non-participation	No participants dropped out of the study or declined to participate once they completed the consent form.
Randomization	For Experiment 1, participants were randomized into either the Low Control or High Control condition.

Reporting for specific materials, systems and methods

We require information from authors about some types of materials, experimental systems and methods used in many studies. Here, indicate whether each material, system or method listed is relevant to your study. If you are not sure if a list item applies to your research, read the appropriate section before selecting a response.

Materials & experimental systems

n/a	Involved in the study	
☒	☐	Antibodies
☒	☐	Eukaryotic cell lines
☒	☐	Palaeontology
☒	☐	Animals and other organisms
☐	☒	Human research participants
☒	☐	Clinical data

Methods

n/a	Involved in the study	
☒	☐	ChIP-seq
☒	☐	Flow cytometry
☒	☐	MRI-based neuroimaging

Human research participants

Policy information about studies involving human research participants

Population characteristics	See above
Recruitment	Participants were recruited online through Amazon Mechanical Turk.
Ethics oversight	Harvard University Committee on the Use of Human Subjects

Note that full information on the approval of the study protocol must also be provided in the manuscript.
